# Intraparotid Neurofibroma of the Facial Nerve: A Case Report

**Published:** 2016-07

**Authors:** Ahmed-Abdel-Fattah Nofal, Mohammad-Waheed El-Anwar

**Affiliations:** 1 *Department of Otorhinolaryngology , Faculty of Medicine, Zagazig University, Egypt. *

**Keywords:** Facial Nerve, Parotid Gland, Neurofibroma

## Abstract

**Introduction:**

Intraparotid neurofibromas of the facial nerve are extremely rare and mostly associated with neurofibromatosis type 1 (NF1).

**Case Report:**

This is a case of a healthy 40-year-old man, which underwent surgery for a preoperatively diagnosed benign parotid gland lesion. After identification of the facial nerve main trunk, a single large mass (6 x 3 cm) incorporating the upper nerve division was observed. The nerve portion involved in the mass could not be dissected and was inevitably sacrificed with immediate neuroraphy of the upper division of the facial nerve with 6/0 prolene. The final histopathology revealed the presence of a neurofibroma. Complete left side facial nerve paralysis was observed immediately postoperatively but the function of the lower half was returned within 4 months and the upper half was returned after 1 year. Currently, after 3 years of follow up, there are no signs of recurrence and normal facial nerve function is observed.

**Conclusion:**

Neurofibroma should be considered as the diagnosis in a patient demonstrating a parotid mass. In cases where it is diagnosed intraoperatively, excision of part of the nerve with the mass will be inevitable though it can be successfully repaired by end to end anastomosis.

## Introduction

Solitary neurofibroma of the facial nerve is generally uncommon and most cases affect the intracranial and intratemporal parts. Intraparotid neurofibromas of the facial nerve are extremely rare and mostly associated with neurofibromatosis type 1 (NF1) (1). In this current study, a case of solitary neurofibroma of the intraparotid part of the facial nerve in a man without NF1was reported.

Preoperative diagnosis of facial nerve neurofibromas in the parotid gland is generally difficult because of the low frequency of the disease and the few typical signs associated with it. The most common presenting symptom is a painless slow growing parotid mass. The estimated incidence of parotid tumors of facial nerve origin ranges from 0.2% to 1.5% (1), with 3.9% of these tumors finally diagnosed as malignant (2).

Nerve preservation during removal of neurofibroma is difficult and nerve reconstruction is usually needed either by end to end anastomosis, nerve grafts, or nerve transfer (3-8).

## Case report

**Fig 1:**
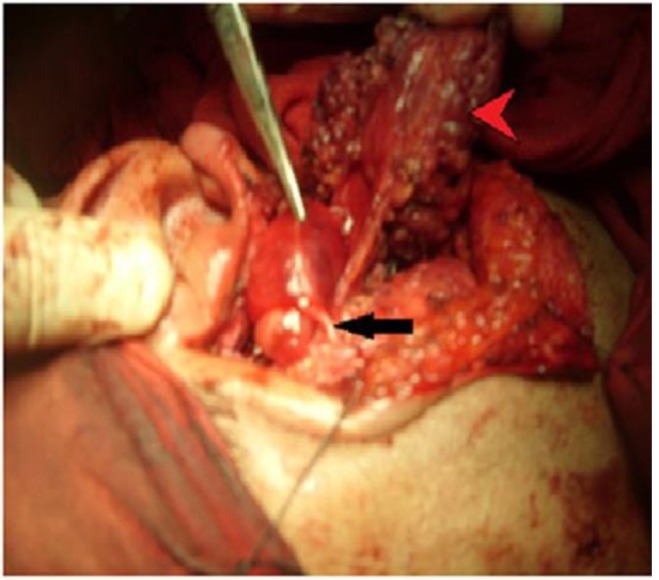
After removal of the superficial part of the parotid gland (red arrow head) a large mass incorporating the superior division of the facial nerve (black arrow) was observed

A healthy 40-year-old man presented with a painless left infra-auricular swelling, which had been present for about 2 years. Clinical examination revealed an oval, firm, non-tender mass, which was not adherent to the overlying skin in the left parotid gland region and which showed normal facial nerve function. No other abnormality was detected in the patient. Computed tomography (CT) revealed a heterogeneously enhancing lesion with cystic necrosis in the left parotid region. Fine needle aspiration cytology (FNAC) revealed a benign lesion with no malignant cells. The lesion was considered as a benign parotid gland lesion. During surgery and after identification of the main trunk of the facial nerve, the superficial part of the parotid gland was started to be removed. A single large mass of 6×3 cm incorporating the upper division of the facial nerve was observed (Fig.1). 

**Fig 2:**
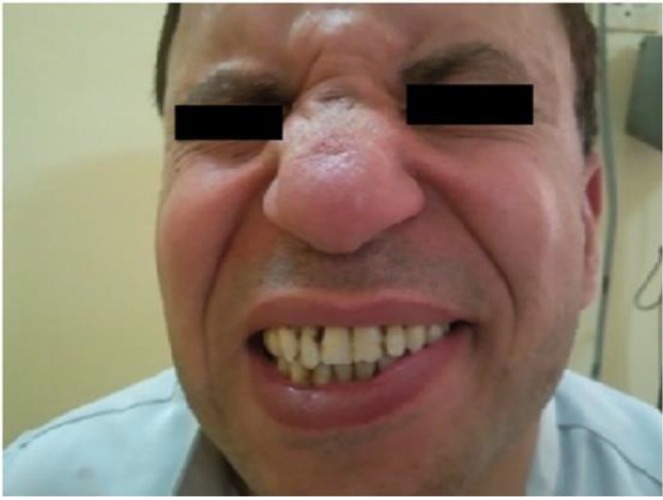
The patient’s one year follow up (post operatively) with recovery of the facial nerve function

Dissection of the mass from the facial nerve failed so the nerve portion, which was involved in the mass (nearly 1.5 cm), was inevitably sacrificed with immediate tensionless neuroraphy of the upper division of the facial nerve with 6/0 prolene. The final histopathology revealed a neurofibroma of the facial nerve. Complete left side facial nerve paralysis was observed immediately postoperatively and, through patient follow up, the function of the lower half was returned within 4 months and the upper half was returned after 1 year (Fig.2). 

Currently, after 3 years of follow up, there are no signs of recurrence with normal facial nerve function.

## Discussion

Primary neurogenic tumors of the facial nerve are uncommon and these tumors, which arise from Schwann cells, include the more common schwannoma while the exceedingly rare neurofibroma mostly affects the intratemporal part compared to the intracranial and intraparotid parts, which are much less affected (9).

Neurofibromas may occur most commonly as a part of Von Recklinghausen’s disease (neurofibromatosis type 1, NF-1) (3,10), which is a relatively common genetic disorder that is inherited in an autosomal dominant pattern with a wide range of expression. The major features of NF-1 include multiple neural tumors, “café au lait” spots, and Lisch nodules (11). The case reported in this current study was single without any detected NF-1 features.

Malignant transformation of neurofibromas is uncommon. Malignant transformation has been reported in 10-15% of plexiform neurofibromas, but it is more common in patients whose neurofibromas arise in the setting of NF-1 and patients with deeply seated neurofibromas (3,12-14). 

Intraparotid neurogenic neoplasm is difficult to be diagnosed correctly preoperatively (3,5,15,16). FNAC and even incisional biopsy can fail to provide a correct diagnosis (3,5,7). Sullivan et al. noted that neurogenic neoplasms of the intraparotid facial nerve are usually only diagnosed intraoperatively by tissue biopsy (3). This was the condition present in this case as no preoperative diagnosis was made but rather an intraoperative suggestion from relation to facial nerve was made and proven through postoperative histopathological examination.

Management of neurogenic tumors of the intraparotid facial nerve is controversial unlike schwannomas which tend to displace nerves and thus allow nerve preservation. Neurofibromas, however, incorporate the nerves and are generally resected en bloc with the involved nerve (18). Therefore, in this case, neurofibroma was removed completely with the involved nerve with successful immediate nerve repair.

## Conclusion

Neurofibroma should be considered as the diagnosis in a patient with a parotid mass even with benign preoperative criteria upon examination, CT, and FNAC. The authors believe that every effort should be done preoperatively in order to diagnose it properly. But in cases in which diagnosis is performed intraoperatively, excision of part of the nerve with the mass will be inevitable; however, immediate repair of the facial nerve could be accomplished by end to end anastomosis with good recovery of function during the course of follow up.
